# A Mask-Assisted Radar Signal Sorting Method Based on Digitized PDW and U1DADM

**DOI:** 10.3390/s26144464

**Published:** 2026-07-14

**Authors:** Yumin Sun, Peng Li, Yingchao Chen, Erxing Yan

**Affiliations:** School of Electronic Engineering, Xidian University, Xi’an 710071, China; sym@stu.xidian.edu.cn (Y.S.); 19021110339@stu.xidian.edu.cn (Y.C.); exyan@stu.xidian.edu.cn (E.Y.)

**Keywords:** radar signal sorting, semantic segmentation, adaptive dilated convolution, focal loss

## Abstract

To address the challenge of effectively sorting radar signals with identical modulation types and overlapping parameter ranges in complex electromagnetic environments using traditional methods, this paper proposes a one-dimensional convolutional neural network, U1DADM, based on semantic segmentation. This network extracts deep semantic information from preprocessed digitized signals and introduces a masking strategy for overlapping pulse regions. The proposed digitized data processing method achieves joint modeling of intra-pulse and inter-pulse features. The core module of the U1DADM network, adaptive dilated convolution, achieves multi-scale feature fusion and long-range feature dependency modeling through dynamic receptive field adjustment. The masking strategy mitigates data defects and assists the network in focusing on repetitive pulse regions. Experimental results indicate that the proposed method surpasses similar deep learning methods and traditional sorting methods under various data conditions. Furthermore, it exhibits strong robustness and generalization ability even under harsh conditions with spurious and missed pulses, achieving high-precision radar signal sorting.

## 1. Introduction

Unlike radar systems that receive and process known target signals, radar reconnaissance systems employ reconnaissance antennas to continuously and omnidirectionally intercept signals emitted from all unknown radar radiation sources (RRS) in the electromagnetic environment. Consequently, the reconnaissance receiver typically receives time domain interleaved pulse streams generated by multiple radar emitters, resulting in severe signal overlap. Based on this, the process of measuring parameters, extracting features and analyzing patterns of various pulses in the received signal, and correctly separating pulses belonging to the same RRS is called radar signal sorting (RSS), also known as pulse stream deinterleaving [1]. RSS is a critical component of electronic support measures (ESMs). It provides an important data source foundation and technical support for subsequent tasks such as modulation identification [2], individual identification [3], working mode identification [4], and behavior identification [5], thereby assisting the combat system in realizing battlefield situation awareness and decision analysis. As a result, the RSS problem has long been extensively studied by researchers worldwide, forming multiple research frameworks, including traditional rule-based methods, clustering methods, and deep learning methods, with many representative achievements reported in the literature [6].

However, with the development of modern radar technologies and the continuous enhancement of anti-reconnaissance capabilities, radar emitter modulation schemes are becoming increasingly complex, while the switching of parameters and working modes is becoming more flexible and dynamic, posing significant challenges to RSS techniques. Therefore, effectively extracting the semantic information contained in radar pulses is particularly important for RSS. Shuo Yuan et al. [7] proposed the semantic coding theory of PRI mode to reveal and reconstruct the PRI mode of intercepted radar pulse stream. Wang Chao et al. [8] used the semantic segmentation method for pulse deinterleaving based on the semantic information hidden in difference of time of arrival (DTOA) data. However, the above methods mainly use inter-pulse information for modeling, and the sorting performance is vulnerable to complex environments and noise interference. Rouxuan Chen et al. [9] introduced the multi-feature semantic deinterlacing (MFSD) method to achieve RSS by mining deep features such as parameter values, modulation modes, and relationships between parameters. Tao Chen et al. [10] mapped pulse description words (PDW) to point cloud data and used a multi-stage graph convolutional network (MSGCN) based on graph convolution to achieve point cloud segmentation and RSS. Although the above methods utilize complete PDW data, pulse amplitude (PA) is easily affected by the distance and transmission power of the RRS, while DOA varies greatly in different scenarios, which limits the generalization capability of the network. In addition, Tao Chen et al. [11] converted PDW into a two-dimensional image with time of arrival (TOA) as the horizontal axis, radio frequency (RF) as the vertical axis, and pulse width (PW) as the color, followed by semantic-segmentation-based RSS. Boyi Yang et al. [12] transformed the signal into a time-frequency spectrogram and combined it with masked instance segmentation to solve the deinterleaving problem caused by pulse overlap. Ji-Hyeon Kim et al. [13] proposed a two-stage RSS framework based on the image of signal amplitude changing over time. Although the above methods can effectively exploit the advantages of image-based networks, converting the originally simple-structured PDW data into two-dimensional images or time-frequency spectrograms introduces a large amount of redundant background information, thereby increasing the consumption of computing resources and model training time.

To address complex scenarios characterized by identical modulation types, overlapping parameter ranges, and highly similar parameter distributions among different RRS, this paper performs data preprocessing using TOA, PW, and RF features and constructs a digitized PDW representation. Based on this, a one-dimensional convolutional neural network named U1DADM is proposed based on semantic segmentation to capture semantic relationships among features and achieve RSS through an encoding-decoding framework. The proposed digital PDW retains complete and stable information and features while standardizing the input data length and effectively mitigating the impact of the randomness and non-stationarity of the original pulse TOA data on network training. Simultaneously, the semantic segmentation concept is introduced into the one-dimensional neural network, allowing the model to leverage pixel-level feature modeling capabilities and jointly learn individual pulse features and global semantic dependencies, thereby enhancing semantic information extraction from the pulse stream. The main contributions of this paper are summarized as follows:

1. A semantic segmentation model, U1DADM, based on a one-dimensional convolutional neural network is proposed for end-to-end deinterleaving of complex radar pulse stream;
2. A mask-assisted training strategy is proposed, which enhances the ability of network to perceive and recognize overlapping pulse regions by introducing a pulse overlap mask;
3. A novel digital preprocessing method is proposed, which translates the interleaved PDW data into digitized PDW, effectively improving the network training stability and feature representation ability.

Experimental results show that in scenarios where different RRS employ the same modulation type and exhibit highly overlapping parameter ranges, the proposed method can comprehensively utilize both intra-pulse and inter-pulse features to achieve more accurate semantic feature modeling, demonstrating superior robustness and generalization capability. Compared with traditional methods and similar deep learning methods, the proposed method exhibits superior sorting performance in complex environments. Even under harsh conditions where both the false pulse rate and pulse loss rate are 20%, the proposed method still achieves a sorting accuracy exceeding 97%.

## 2. Related Work

### 2.1. Radar Signal Sorting

Research on RSS can be traced back to the 1970s, and most traditional methods are based on template matching [14]. Specifically, the main features and parameter information of a large number of radar emitters are collected in advance and stored in a database as templates. The parameters of received signals are then compared with the template feature parameters to determine whether the received signals originate from the same radar emitter. Although this method can obtain relatively ideal sorting results in a short time, it relies heavily on prior knowledge. Since the relationship between the reconnaissance side and the radar side is non-cooperative, acquiring prior knowledge is highly challenging. Moreover, the method is prone to false alarms and missed detections for targets that do not exist in the database. Since the PRI parameter is relatively stable compared with other parameters, numerous PRI-based RSS methods have been developed, such as the PRI search method [15], PRI histogram method [16,17], PRI transformation method [18], and plane transformation method [19]. However, these methods become ineffective when PRI variations are highly complex or when the pulse stream density is high.

With the rapid development of artificial intelligence, many new methods have emerged in the field of RSS. For example, unsupervised clustering methods such as K-means [20], grid clustering, and density clustering [21,22] are represented. Although this method is flexible and fast, it has high requirements for data shape and parameter settings. Supervised classification-based neural networks are also among the most popular research approaches for RSS. Support Vector Machine (SVM) [23] and Long Short-Term Memory (LSTM) networks achieve substantially better performance than conventional methods.

### 2.2. Semantic Segmentation

Semantic segmentation is a core task in the field of computer vision, aiming to classify each pixel in an image to accurately label the specific location and shape boundary of each object in the image. The concept of semantic segmentation originated from early machine learning methods based on image segmentation such as region growing, threshold segmentation, and edge detection. In 2006, Jamie Shotton et al. [24] first combined image segmentation with semantic categories in the TextonBoost framework and formally introduced the concept of semantic segmentation.

With the development of deep learning, semantic segmentation has achieved significant breakthroughs, and a series of milestone methods have emerged. Long et al. [25] first realized end-to-end prediction from image to label based on a Fully Convolutional Network (FCN). Subsequently, convolutional neural networks (CNNs) such as U-Net [26], SegNet [27], and DeepLab [28] further enhanced the capability of modeling complex scenes and significantly improved segmentation accuracy. More recently, attention-based architectures such as Vision Transformer (ViT) [29] have also been introduced into semantic segmentation, demonstrating stronger long-distance dependency modeling ability. In addition, hybrid methods, CNN networks, and Transformers [30,31] have provided new perspectives for achieving more robust and generalized image understanding.

Overall, semantic segmentation, with its pixel-level modeling capabilities and methodological effectiveness, provides a new solution for the field of RSS.

### 2.3. Focal Loss

Focal Loss is an improved cross-entropy loss function proposed by Lin et al. [32] in 2017 and was first applied in the single-stage object detection framework RetinaNet. This method aims to solve the problem of class imbalance. Its core idea is to introduce a modulation factor and a class balancing factor into the loss function, thereby assigning different category weights to the model during training to reduce the contribution of easily classified samples to the overall loss and emphasize the importance of hard samples and foreground classes. This method effectively alleviates the training difficulties caused by the extreme imbalance between foreground and background samples in single-stage detectors and for the first time makes single-stage detectors comparable to two-stage methods in terms of accuracy.

Since its introduction, Focal Loss has been widely applied and extended in various fields beyond object detection. For example, it has been used to alleviate class imbalance problems in tasks such as semantic segmentation and image classification, achieving remarkable performance improvements. Furthermore, several improved variants of Focal Loss have been proposed for specific tasks and more complex scenarios. For example, Zehuan Jing et al. [33] introduced an adaptive Focal Loss into intra-pulse modulation recognition for few-shot radar pulses, significantly improving classification performance under complex backgrounds. Tal Ridnik et al. [34] improved Focal Loss to obtain Asymmetric Loss in multi-label classification tasks, which can dynamically reduce the weight of easy samples while discarding samples that may be mislabeled. These methods further enhance model robustness under complex data distributions.

In summary, Focal Loss is an effective tool for addressing class imbalance and has become an important branch of research on loss functions in deep learning.

## 3. Proposed Method

### 3.1. Overall Structure

**Figure 1:**
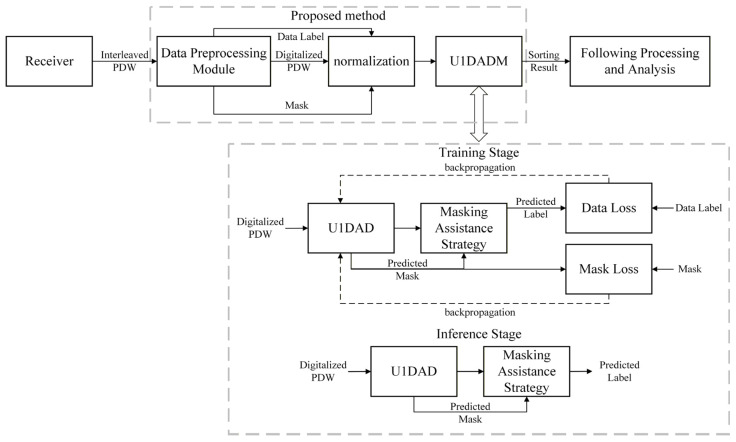
The overall structure of the proposed method.

The overall structure of the proposed method is shown in Figure 1. The system takes interleaved PDW data as input and outputs the corresponding radar pulse labels. First, the interleaved PDW data are sent to the data preprocessing module, where digitized encoding is performed according to the original pulse TOA parameters, and data labels and mask information for identifying pulse overlap regions are generated simultaneously. The digitization process converts PDW data of varying lengths into a fixed-length suitable for neural network input, while the mask is used to describe the overlap of different pulses during the digitization mapping process. Subsequently, the PW and RF parameters in the digitized PDW data are normalized to reduce the influence of parameter scale differences on network training. During the training stage of the proposed U1DADM sorting network, the digitized PDW data, the corresponding data labels, and the masks are jointly used for network training. The network takes the digitized PDW as the input and simultaneously outputs the predicted mask and the predicted labels under the assistance of the predicted mask. The predicted mask and predicted labels are then compared with the corresponding ground-truth mask and ground-truth labels to compute the respective loss functions, which are used to supervise and optimize the learning of both mask prediction and pulse classification. During the inference stage, only the digitized PDW data are required as the network input, without the need for ground-truth labels and overlap masks. The network first predicts the overlap mask directly from the input digitized PDW and then performs the proposed masking assistance strategy based on the predicted mask to generate the final labels, thereby obtaining the final RRS sorting results.

U1DADM is an abbreviation for the network architecture and masking assistance strategy proposed in this paper. The network is a one-dimensional RSS network developed based on the classic U-Net structure. To address the problems of low effective pulse region ratio, large span of effective information between pulses, and the difficulty in extracting long-range correlated features in digitized PDW data, an adaptive dilated convolution (ADC) module is introduced into the network. By dynamically adjusting the receptive field, the proposed module enhances the network’s ability to extract multi-scale features and long-range feature dependencies, thereby improving feature representation performance for complex pulse stream.

Furthermore, considering that pulse overlap is likely to occur during the digitized mapping process of PDW data, which may lead to RRS pulse loss and information corruption, a mask-assisted strategy is further introduced in this paper. This strategy guides the network to perceive and identify pulse overlap regions and generate the corresponding overlap masks, thereby enhancing the model’s discrimination capability for overlapping pulses and alleviating the degradation in sorting performance caused by pulse overlap or loss during the digitized preprocessing stage. Through the collaborative effect of ADC and the masking mechanism, the U1DADM network can simultaneously exploit inter-pulse temporal relationships and intra-pulse parameter features, achieving effective RSS in complex electromagnetic environments.

### 3.2. Data Preprocessing

Typically, after interception by the reconnaissance receiver, RSS that can be easily distinguished are preliminarily classified according to their channel information and DOA from the perspective of physical characteristics, thereby reducing the computational complexity of subsequent fine sorting and mitigating mutual interference among pulses. Then, the received pulse stream is framed over a selected time interval to reduce the number of pulses processed in a single operation and alleviate the burden of subsequent sorting. Nevertheless, PDW data consist of multidimensional parameters, such as TOA, PW, RF, PA, and DOA, which exhibit significant differences in physical meanings, statistical characteristics, and variation patterns. Therefore, directly feeding PDW data into the network not only makes it difficult to fully exploit the feature representation capabilities of different parameters but may also introduce redundant information and interference, which negatively affects sorting performance.

**Figure 2:**
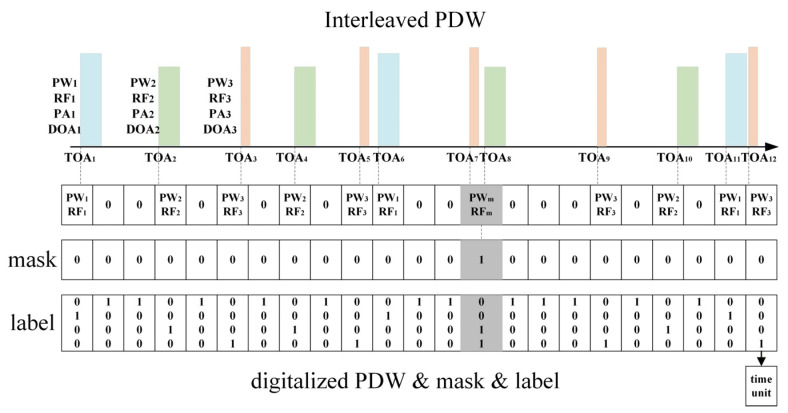
Interleaved PDW is preprocessed into digitalized PDW, mask, and label.

To address the above issues, a dedicated data preprocessing method is designed in this paper for single-frame pulse data, as shown in Figure 2, which realizes the transformation from interleaved PDW to digitized PDW representations. Since the pulse arrival times and pulse combinations are random, but the duration of each data frame is fixed, the number of pulses contained in different data frames is variable. The random variation in pulse number leads to instability in the dimensionality and distribution of the input data, which is unfavorable for feature learning and modeling by neural networks. Furthermore, the TOA sequence continuously increases over time, exhibiting non-stationarity and non-convergence characteristics. Directly feeding the original data into the network may result in unstable network training or even convergence difficulties. Based on these two considerations, the TOA information is first digitized into a fixed-length discrete representation and introduced a mask to represent the pulse overlap region. This process not only unifies the input data structure, but also enhances the model’s adaptability to noise interference, parameter jitter, and pulse overlap conditions. On this basis, PW and RF information are further fused to jointly construct the network input data, thereby preserving the inter-pulse temporal relationship while introducing intra-pulse parameter features. By jointly modeling both intra-pulse and inter-pulse information, the proposed method not only effectively improves the completeness of radar emitter feature representation but also enhances the network’s capability to distinguish overlapping pulses and pulses with similar parameters in complex pulse streams, thereby improving network robustness and overall pulse sorting performance.

Specifically, a frame of data containing K pulses is represented as $S_{1}=[s_{1},s_{2},\ldots ,s_{K}]$, and an individual pulse is represented as $s_{k}=[t_{k},p_{k},f_{k},a_{k},d_{k}],1\leq k\leq K$. The TOA sequence is denoted as $t=[t_{1},t_{2},\ldots ,t_{K}]$, the PW sequence as $p=[p_{1},p_{2},\ldots ,p_{K}]$, the RF sequence as $f=[f_{1},f_{2},\ldots ,f_{K}]$, the PA sequence as $a=[a_{1},a_{2},\ldots ,a_{K}]$, and the DOA sequence as $d=[d_{1},d_{2},\ldots ,d_{K}]$. The time duration L of a frame is divided into multiple time units $u_{t}$. If a pulse exists within a certain time unit, the PW and RF parameters of that pulse are assigned to that time unit. If multiple pulses exist within the same time unit, the average of each parameter is assigned. Time units without pulses are assigned a value of 0. This process is analogous to the sampling process in digital signal processing. Finally, the interleaved PDW data is mapped into the digitized PDW data $X_{1}=[x_{1},x_{2},\ldots ,x_{N}]\in {\mathbb{R}}^{2\times N}$, where $N=L/u_{t}$ is the number of time units in a frame of data. The mathematical expression for the above process is as follows:

(1) $$x_{n}=\left\{\begin{array}{ll} (p_{i},f_{i}), & \left|I_{n}\right|=1\\ (p_{m},f_{m}), & \left|I_{n}\right|>1\\ (0,0), & \mathrm{otherwise} \end{array}\right..$$

where $I_{n}=\{i\in \{1,\ldots ,K\}\left|nu_{t}\right.<t_{i}\leq (n+1)u_{t}\},1\leq n\leq N$ is the index set, and $p_{m},f_{m}$ is the average value of the PW and RF parameters in the time unit. To identify time units with overlapping pulses, a mask vector $m=[m_{1},m_{2},\ldots ,m_{N}]\in {\mathbb{R}}^{1\times N}$ is defined. If there are multiple pulses in a time unit, it is marked as 1; otherwise, it is marked as 0. The mathematical formulation of the above process is expressed as follows:

(2) $$m_{j}=\left\{\begin{array}{ll} 1, & \left|I_{j}\right|>1\\ 0, & \mathrm{otherwise} \end{array}\right..$$

The final label is represented as $Y_{1}=[y_{1},y_{2},\ldots ,y_{N}]\in {\mathbb{R}}^{c\times N}$, where c is the category, containing a background and the number of RRS to be sorted. A label value of 1 indicates belonging to the category, and a label of 0 indicates not belonging to it. For non-overlapping time units with a mask value of 0, one-hot labels are adopted, whereas for overlapping time units with a mask value of 1, multi-hot labels are used.

### 3.3. U1DAD

**Figure 3:**
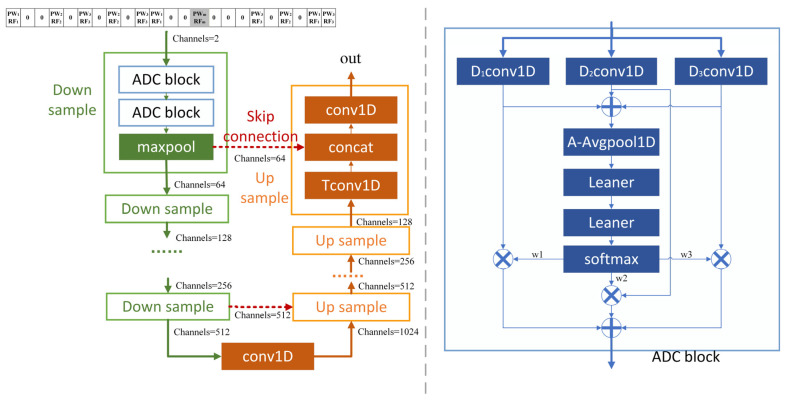
The architecture of U1DAD.

The architecture of U1DAD is shown in Figure 3. The network adopts a symmetrical encoder-decoder structure with skip connections introduced between corresponding layers. The proposed network aims to fully exploit the intrinsic temporal patterns and long-range feature dependencies in radar pulse streams through hierarchical feature extraction and multi-scale feature fusion, thereby achieving end-to-end RSS. The core components of the network consist of an ADC module, a down-sampling module, and an up-sampling module.

Among them, the ADC module serves as the core component of the proposed network. Since the digitized PDW contains extensive zero-valued background regions with sparse effective pulse information, the traditional CNNs are constrained by limited receptive fields and thus have difficulty modeling long-range pulse dependencies. Expanding the receptive field solely through standard convolutions requires deeper network architectures, which significantly increases computational complexity and may cause gradient propagation difficulties as well as reduced training efficiency. To address this issue, dilated convolution [35] is introduced to expand the receptive field by inserting holes in the convolution kernel, thereby improving long-range feature extraction capability without significantly increasing the number of parameters. If the feature of the input layer l is $H^{l-1}\in {\mathbb{R}}^{C_{in}\times L}$ ($H^{0}=X_{n}\in {\mathbb{R}}^{2\times N}$), the n-th element of the j-th output channel generated by the one-dimensional convolutional layer and the one-dimensional dilated convolutional layer can be formulated as follows:

(3) $$h_{j}^{l}(n)=\sum_{i=1}^{C_{in}}\sum_{k=1}^{K}h_{i}^{l-1}(n\cdot S+k-1)\cdot w_{i,j}^{l}(k)+b_{j}^{l},$$

(4) $$h_{j}^{l}(n)=\sum_{i=1}^{C_{in}}\sum_{k=1}^{K}h_{i}^{l-1}(n+r\cdot (k-1))\cdot w_{i,j}^{l}(k)+b_{j}^{l}.$$

where K is the kernel size, S is the stride, r is the dilation factor, $w_{i,j}^{l}\in {\mathbb{R}}^{K}$ is the kernel weight connecting the i-th input channel and the j-th output channel in layer, and $b_{j}^{l}$ is the bias term of the j-th kernel.

Furthermore, considering that the number of pulses varies greatly in different scenarios and that pulse arrivals exhibit strong randomness, the distributions of effective features are inconsistent among different data frames. Consequently, the dilated convolutions with fixed dilation are insufficient to adapt to the complex and varying characteristics of pulse streams. Based on this, an ADC structure is introduced based on the aforementioned one-dimensional dilated convolution. By dynamically adjusting the dilation rates, the network can adaptively match the appropriate receptive field according to the different pulse data characteristics, thereby enhancing adaptability and feature representation capability of the model. The ADC module adopts the adaptive fusion mechanism in the style of Selective Kernel (SK-Net) style [36]. Specifically, the input $H^{l-1}$ is first fed into B one-dimensional dilated convolution branches with different dilation factors $r_{b}$ to obtain a set of feature matrices $\left\{H_{1},H_{2},\ldots ,H_{B}\right\}$. Then, adaptive weights are generated by the feature aggregation and selection network. Finally, the j-th channel of the fused output F is:

(5) $$f_{j}^{l}=\sum_{b=1}^{B}w_{b}\cdot h_{b,j}^{l}.$$

where $w_{b}$ is the adaptive weight of the b-th branch, and its generation process is described as follows:

(6) $$w_{b}=\mathrm{softmax}{\left(\mathrm{FC}(z)\right)}_{b}=\frac{\exp({\mathrm{FC}}_{b}(z))}{\sum_{m=1}^{B}\exp({\mathrm{FC}}_{m}(z))}.$$

Here, $z\in {\mathbb{R}}^{C_{out}}$ is the channel-level global descriptor, which is obtained by first summing the element-wise outputs of all branches to get the aggregated features $U=\sum F_{j},U\in {\mathbb{R}}^{C_{out}\times L}$, and then extracting them through global average pooling. The calculation process for one channel is as follows:

(7) $$z_{j}=\frac{1}{L}\sum_{n=1}^{L}u_{j}(n).$$

The down-sampling module is used to further expand the network receptive field and extract abstract semantic information in a higher-level feature space. By compressing the feature dimensions layer by layer, the network enhances its capability to model global pulse distribution patterns and long-range dependency features. The ADC module and the down-sampling module together constitute the encoder, enabling hierarchical encoding and deep semantic extraction of the input pulse stream features.

The decoder mainly consists of up-sampling modules, which are used to gradually recover feature resolution and pulse position information. Since the down-sampling process inevitably leads to the loss of certain local spatial details, skip connections are introduced between the encoder and decoder to fuse shallow high-resolution features with deep semantic features. This effectively compensates for the loss of detailed information and enhances the network’s ability to preserve local single-pulse features.

Through the above architectural design, the U1DAD network can not only learn the global semantic features and long-range dependencies in complex pulse streams but also effectively preserve local single-pulse detail information, thereby improving the accuracy and robustness of RSS in complex electromagnetic environments where different emitters employ identical modulation types and exhibit similar parameter ranges.

### 3.4. Masking Assistance Strategy

**Figure 4:**
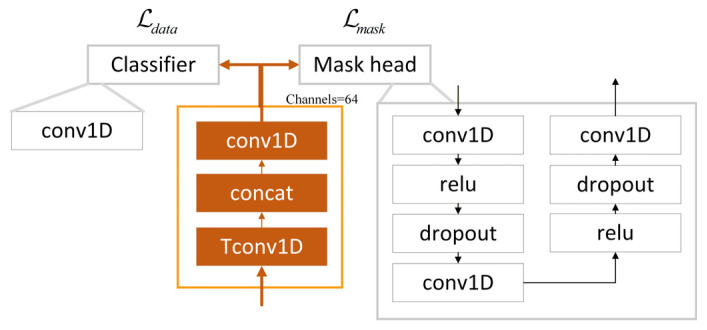
The mask-assisted module.

Since multiple pulses may fall into the same time unit after PDW digitization, a mask mechanism is introduced to compensate for the limitations of the preprocessing procedure by guiding the network to focus on these time units. Accordingly, a mask-assisted module is appended to the end of the proposed network, as shown in Figure 4, enabling the network to learn and represent the mask pattern of each frame of data and subsequently assist the sorting process.

Specifically, regions with mask values of 1 are processed using multi-label classification, whereas regions with mask values of 0 are processed using single-label classification. Instead of applying multi-label classification to all samples, the proposed mask-assisted strategy separately processes different regions to reduce the influence of background noise and false pulses on sorting accuracy, thereby enhancing the robustness and anti-interference capability of the network.

The loss function for the mask uses a weighted binary cross-entropy loss function:

(8) $${ℒ}_{mask}=-\frac{1}{N}\sum_{n=1}^{N}\left[w\cdot m_{n}\log(\hat{m_{n}}\right)+(1-w)(1-m_{n})\log(1-\hat{m_{n}})].$$

In the formula, $m_{n}\in \{0,1\}$ represents the true label of the mask at the n-th pixel; $\hat{m_{n}}\in [0,1]$ is the predicted probability of the pixel belonging to the corresponding class, which is obtained by applying the sigmoid activation function [37] to the network output, and the value ranges from 0 to 1; w is the weighting factor, which is used to alleviate sample imbalance.

For RSS, different activation functions are first selected according to the mask information learned by the network. Specifically, when the current time unit is located in an overlapping region ($m_{n}=1$), the sigmoid function is employed to calculate the independent probability of each class; when the current time unit belongs to a non-overlapping region ($m_{n}=0$), the softmax function [38] is used to compute the category distribution. Let the segmentation feature output of the model be represented as $p_{n}\in {\mathbb{R}}^{C}$, where C is the number of classes; then the predicted probability $\hat{y_{n}}\in {\left[0,1\right]}^{c}$ of this time unit is defined as follows:

(9) $$\hat{y_{n}}=\left\{\begin{array}{ll} \mathrm{sigmoid}(p_{n})=\frac{1}{1+\exp(-p_{n})}, & \mathrm{if}\;m_{n}=1\\ \mathrm{softmax}{\left(p_{n}\right)}^{\left(c\right)}=\frac{\exp(p_{n}(c))}{\sum_{j=1}^{C}\exp(p_{n}(j))}, & \mathrm{if}\;m_{n}=0 \end{array}\right..$$

Furthermore, due to the sparsity of effective information in digitized PDW data, the focal loss proposed by Lin et al. in 2017 [32] is adopted as the loss function. Its core idea is to introduce a class balancing factor and a focusing factor into the loss function and assign different class weights to the model during training to reduce the contribution of easily classified samples to the overall loss, thereby highlighting the importance of difficult-to-classify samples and foreground classes.

(10) $${ℒ}_{data}=\sum_{n=1}^{N}\sum_{c=1}^{C}\left[-\alpha \cdot {\left(1-{\hat{y}}_{n}^{\left(c\right)}\right)}^{\gamma }\cdot y_{n}^{\left(c\right)}-(1-\alpha )\cdot {\left({\hat{y}}_{n}^{\left(c\right)}\right)}^{\gamma }\cdot (1-y_{n}^{\left(c\right)})\log(1-{\hat{y}}_{n}^{\left(c\right)})\right].$$

In the formula, ${\hat{y}}_{n}^{\left(c\right)}$ represents the predicted probability that the pulse contained in the n-th time unit belongs to the c-th class, $y_{n}^{\left(c\right)}\in \{0,1\}$ is the label corresponding to the time unit, the balance factor α∈(0,1) is the class weight adjustment factor, and the focusing factor γ≥0 is the weight adjustment factor for easy and difficult samples.

During training, the pulse classification task and the mask prediction task are optimized using an alternating optimization strategy. Specifically, the network simultaneously predicts pulse categories and overlap masks from the input data. First, the mask loss is computed using the predicted mask and the corresponding ground-truth mask, and only the parameters of the mask prediction branch are updated through backpropagation. Subsequently, the predicted mask is used as auxiliary information for pulse classification. The classification loss is then calculated based on the predicted pulse categories and the corresponding ground-truth labels, after which only the parameters associated with the pulse classification branch are updated. The two optimization processes are performed alternately until the network converges.

Compared with direct joint optimization, the proposed alternating optimization strategy offers two major advantages. First, it fully exploits the auxiliary role of the mask prediction task in pulse classification, enabling the network to focus more effectively on pulse overlap time units. Second, since the two tasks have different optimization objectives, joint backpropagation may lead to gradient conflicts, thereby degrading convergence speed and overall model performance. By alternately optimizing the two tasks, gradient interference is effectively alleviated, allowing mask prediction and pulse classification to complement each other and ultimately improving the convergence stability and sorting performance of the network.

## 4. Experiment

### 4.1. Experimental Setup

#### 4.1.1. Parameter Settings

The data generation platform was MATLAB R2023a; the deep learning runtime platform was PyCharm 2024; the operating system was Windows 11; and the hardware environment included an Intel Core i7 processor and an NVIDIA GeForce RTX 4060 graphics card.

The deep learning framework used for training and predicting is PyTorch 2.6.0. The network optimizer is Adam, with an initial learning rate of 0.001. To prevent training oscillations caused by an excessively large learning rate or convergence stagnation caused by an overly small learning rate, the ReduceLROnPlaten scheduling strategy [39] is employed. Specifically, the learning rate is automatically decreased when the validation metric shows no improvement within five consecutive epochs, thereby improving convergence stability and overall model performance. Furthermore, an early stopping strategy is utilized to alleviate overfitting during training. Following the original focal loss paper [32], the balancing factor and focusing factor are set to 0.25 and 2, respectively.

#### 4.1.2. Evaluation Indicators

This paper uses sorting accuracy, mask accuracy, sorting over-grouping rate (SOGR), sorting under-grouping rate (SUGR), false alarm rate (FAR), and missed alarm rate (MAR) to evaluate the performance of RSS. A value of 1 indicates that a pulse belongs to the corresponding class, whereas a value of 0 indicates that it does not belong to the class. The confusion matrix is shown in Table 1.

The sorting accuracy refers to the proportion of radar pulse signals whose categories are correctly predicted. It is defined as follows, where n is the number of RRS.

(11) $$ACC_{sorting}=\frac{1}{n}\sum_{i=1}^{n}acc_{i},n>1,$$

(12) $$acc_{i}=\frac{TP}{TP+FN}.$$

Mask accuracy is defined as:

(13) $$ACC_{mask}=\frac{TP}{TP+FN}.$$

The traditional definition of the SOGR is the ratio of the number of additional sorted clusters to the actual number of RRS. For example, if there are N radar emitters in the environment, and after clustering and sorting, there are M targets, then the SOGR is:

(14) $$P_{\mathrm{SOGR}}=\frac{M-N}{N}\;M>N.$$

The traditional definition of the SUGR is the ratio of the number of missed radar emitters to the actual number of radar emitters. Specifically, if N radar emitters exist in the environment and M targets are obtained after clustering and sorting, the SUGR is:

(15) $$P_{\mathrm{SUGR}}=\frac{N-M}{N}\;M<N.$$

The FAR refers to the situation where no pulse signal is present but the model incorrectly detects a pulse signal, corresponding to a false positive (FP) in the confusion matrix; the MAR represents the proportion of actual pulse signals that are not detected by the model, corresponding to a false negative (FN).

### 4.2. Dataset Description

The experimental environment used for PDW simulation data generation and data processing was MATLAB R2023a. The simulation data consisted of single-channel data from a reconnaissance receiver after channelization processing using a polyphase filter bank, with a channel bandwidth of 50 MHz. Five RRS were simulated in total. Each RRS adopts the dwell and switch inter-pulse modulation mode, in which the parameter values of each pulse group are randomly selected and combined from predefined parameter tables to emulate the dynamic variation of radar parameters under different mission requirements and working mode transitions. For example, Radar 1 has eight candidate values for both PW and RF, resulting in a total of 64 possible PW–RF parameter combinations. During the simulation, these parameter combinations are randomly switched to mimic the uncertainty and randomness of agile radar parameter variations. Meanwhile, the parameter tables of different RRS are designed to partially overlap, such that different emitters share similar parameter values in one or more dimensions. This configuration constructs a challenging sorting scenario in which different RRS employ identical modulation schemes while exhibiting overlapping parameter ranges. According to engineering experience, the PRI is set to ten times the PW, and the number of pulses in each pulse group is determined by the ratio of the coherent processing interval (CPI) to the PRI. In this work, the CPI is set to 0.00436 s.

Detailed PW and RF settings of each RRS are provided in Table 2. The dataset generation process consists of 24 s of training data, 8 s of validation data, and 8 s of test data. All data are segmented into 10 ms frames, resulting in 2400, 800, and 800 frames for training, validation, and testing, respectively. During data preprocessing, the time unit is set to 5μs, and 2000 points are obtained by digitizing the data sampled from each 10 ms data frame.

To simulate the effects of transmission environments and distortions introduced by receiver hardware, statistical error models are established for TOA, PW, and RF based on the ideal data. Different levels of measurement deviations are then introduced to construct radar pulse datasets that more closely resemble practical electromagnetic environments.

The deviation of TOA mainly originates from environmental noise, clock jitter, and receiver measurement errors. Therefore, the TOA deviation is modeled as zero-mean Gaussian noise. The deviation of PW is primarily caused by waveform distortion induced by the propagation environment (e.g., multipath effects and environmental noise) as well as receiver measurement errors. Accordingly, the PW deviation is modeled using a log-normal distribution. The deviation of RF mainly arises from thermal noise, phase noise, propagation-induced noise, and receiver measurement errors. Therefore, the RF deviation is modeled as the superposition of an absolute Gaussian error and a relative Gaussian error, where the absolute error represents the frequency offset introduced by receiver hardware, while the relative error characterizes the practical phenomenon that the measurement error increases with the carrier frequency.

A total of seven datasets are generated for the experiments. The measurement deviations in Dataset 1 to Dataset 5 increase progressively, whereas Dataset 6 and Dataset 7 contain combined measurement deviations. The standard deviations of the TOA and RF deviations for each dataset are listed in Table 3. Since the variation trend of the PW deviation is consistent with that of the TOA deviation, and the PW deviation is generally slightly larger than the TOA deviation in practice, the standard deviation of the PW deviation is set to $\sqrt{2}$ times that of the TOA deviation.

### 4.3. Ablation Experiments

**Figure 5:**
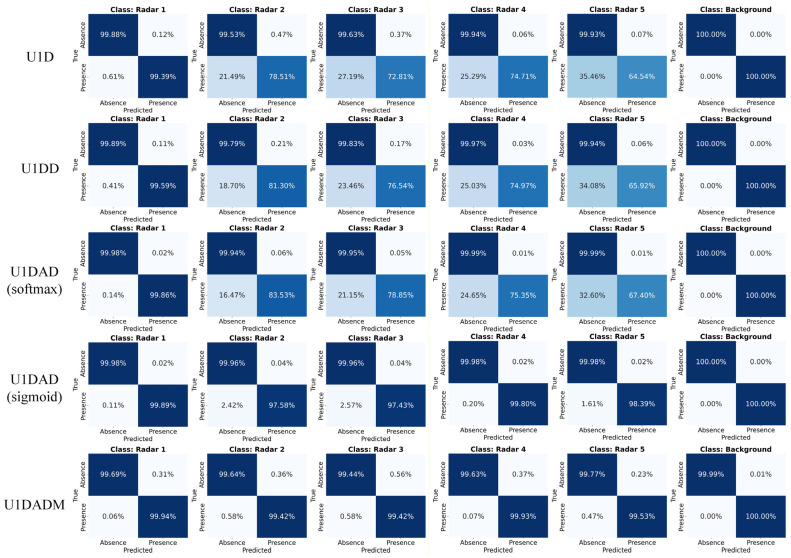
Binary classification confusion matrices based on different methods.

This experiment aims to verify the necessity and effectiveness of each module in the proposed network architecture. The experiment is based on the U1D network, which is a one-dimensional network structure derived from the classic semantic segmentation algorithm U-Net to adapt to one-dimensional pulse data processing tasks. Based on U1D, the U1DD network is constructed by introducing dilated convolution to enlarge the receptive field. Subsequently, ADC is introduced to obtain the U1DAD network to improve multi-scale feature extraction capability and dynamic modeling ability for different features. Finally, the masking assistance strategy is added to the U1DAD network to construct the U1DADM network, enhancing the network’s ability to perceive and separate overlapping pulse regions. The ablation experiments were conducted using Dataset 1 described in Section 4.2, which has a pulse overlap rate of 27.29%. The U1D and U1DD networks are trained using one-hot labels with the cross-entropy loss. For the U1DAD network, two supervision strategies are evaluated. The first employs one-hot labels with the corresponding cross-entropy loss, while the second adopts multi-hot labels with the corresponding binary cross-entropy loss. Based on the U1DAD network, the U1DADM network further introduces the proposed masking assistance strategy and jointly optimizes the pulse classification and mask prediction tasks using an alternating optimization strategy. The corresponding results are presented in Table 4 and Figure 5.

The table records the average sorting accuracy of different networks for five radar emitters. According to the experimental results, each newly introduced component in the U1DADM network contributes to performance improvement. Among them, the introduction of multi-hot labels and the masking assistance strategy yield the most significant improvement in sorting accuracy. This improvement is primarily attributed to its ability to overcome the inherent limitation introduced by the PDW digitization process. Specifically, during the mapping from interleaved PDW to digitized PDW, multiple pulses may be mapped to the same time unit. Conventional one-hot labels assign only a single category to each time unit and therefore cannot accurately represent overlapping pulses, resulting in incomplete supervision. By contrast, multi-hot labels enable all overlapping pulses within the same time unit to be correctly annotated, thereby effectively compensating for this limitation of digitized PDW and substantially improving the network’s ability to recognize overlapping pulses.

However, when only multi-hot labels together with the Binary Cross-Entropy (BCE) loss are employed, the network treats each category as an independent binary classification task. Although this strategy is capable of handling overlapping pulses, it ignores the competition among different radar categories, leading to inferior discriminative capability in non-overlapping regions. To address this issue, a masking assistance strategy is further proposed. Specifically, the Softmax activation together with the cross-entropy loss is adopted for non-overlapping regions to enforce inter-class competition and enhance category discrimination, whereas the Sigmoid activation together with the BCE loss is employed for overlapping regions to support multi-label prediction. By combining the discriminative advantage of Softmax in single-label classification with the representational capability of Sigmoid in multi-label classification, the proposed strategy effectively handles both non-overlapping and overlapping pulses, thereby achieving the highest sorting accuracy.

The figure further presents the binary confusion matrix of the five radars and the background for different networks. As shown in the figure, with the progressive optimization and refinement of the network architecture, the sorting accuracy of each radar improves to varying degrees, but the background maintains a consistently high accuracy. This is because the background regions with zero-value information are much easier to distinguish compared with the complex radar pulse parameters. Furthermore, although the accuracy of each radar emitter improves, the FAR and MAR do not increase correspondingly, so the proposed network maintains stable performance. Further analysis reveals that radar 5 has the lowest accuracy because its PW and corresponding PRI are relatively larger than those of the other radar emitters, resulting in fewer pulses within the same time period and consequently increasing the learning difficulty.

The experimental results demonstrate that the proposed U1DADM network in this study effectively solves the problems of overlapping pulse parameter ranges and temporal pulse occlusion by introducing ADC to dynamically match receptive fields with multi-scale features and using the masking assistance strategy to identify overlapping pulses.

### 4.4. Comparative Experiment

**Figure 6:**
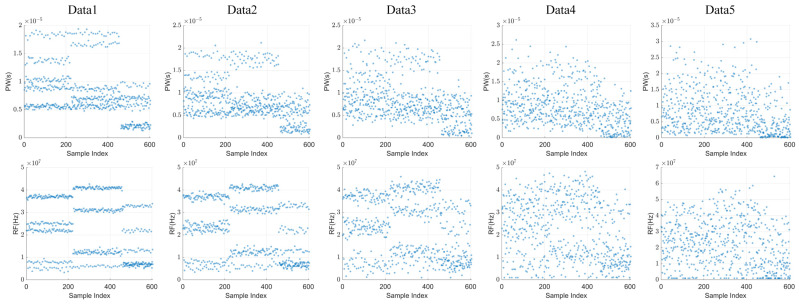
The distributions of the relevant PW and RF under different measurement errors.

To further verify the effectiveness of the proposed method in scenarios with overlapping parameter ranges and pulse overlap, comparative experiments were conducted on the five datasets with progressively increasing measurement errors constructed in Section 4.2. The proposed method was compared with several representative approaches in the same field, including the deep learning-based methods TCN [40], transformer [41], BLSTM [8], and Denoising Autoencoder [42], as well as the classical RSS algorithms Improved K-means [20], DBSCAN [22], and SDIF [17]. The comparison was made in terms of sorting accuracy, SOGR, and SUGR. The distributions of the relevant PW and RF under different measurement errors are illustrated in the corresponding Figure 6.

To ensure a fair comparison, each method employs the data representation that best matches its algorithmic characteristics. Specifically, the proposed U1DADM, TCN, and Transformer networks all adopt the proposed data preprocessing method, which jointly models inter-pulse and intra-pulse features. This enables the networks to fully exploit complementary information from both feature types while alleviating the adverse effects of noise and information loss on sorting performance. The BLSTM method takes the first-order difference of TOA (DTOA) as the input and therefore learns only inter-pulse temporal features. The Denoising Autoencoder employs digitized PDW data containing only TOA information, without intra-pulse parameters such as PW and RF; consequently, its input features also capture only inter-pulse characteristics. Among the conventional sorting methods, the improved K-means and DBSCAN algorithms perform clustering based on the PW and RF parameters in the PDW data, whereas SDIF performs pulse sorting solely according to the TOA information. To further verify the effectiveness of joint modeling of inter-pulse and intra-pulse information and to demonstrate the advantages of the proposed method in complex environments, additional comparative experiments were conducted on Dataset 6 and Dataset 7 with combined measurement errors constructed in Section 4.2. The corresponding experimental results are presented in Table 5 and Table 6. And their computational complexity is shown in Table 7.

It should be noted that the SOGR reflects the extent to which pulses originating from the same RRS are incorrectly divided into multiple groups, whereas the SUGR measures the extent to which pulses from different RRS are erroneously merged into the same group. Therefore, these two metrics are adopted to evaluate RSS performance from two complementary perspectives, namely, over-segmentation caused by intra-emitter parameter agility and under-segmentation caused by inter-emitter parameter similarity. To ensure a fair comparison, only three RRS are selected to construct the evaluation subset when calculating SOGR and SUGR. This setting avoids the evaluation bias introduced by the fixed output categories of the closed-set classification models, including U1DADM, TCN, and Transformer. For conventional clustering methods, parameter similarity among different RRS may cause multiple radars to be merged into a single cluster, while parameter agility within the same radar may cause its pulses to be divided into multiple clusters. Similarly, for the closed-set neural network models, although the test set contains only three radar emitters, the prediction space still consists of all five training categories. Consequently, pulses from the same RRS may be incorrectly assigned to different training categories, resulting in additional radar groups. Conversely, pulses from different RRS may also be assigned to the same predicted category, leading to the erroneous merging of multiple radars. Therefore, both conventional clustering methods and closed-set neural network models may exhibit sorting over-grouping and sorting under-grouping phenomena.

Analysis of the experimental results indicates that as measurement error increases, the PW and RF parameter features gradually become discrete, and the parameter boundaries become increasingly blurred. Consequently, the sorting accuracy of most methods decreases to varying degrees. However, deep learning methods exhibit stronger robustness than clustering methods, among which U1DADM is affected the least by interference. This demonstrates that deep learning methods are capable of extracting stable and discriminative feature representations from disordered data for RSS, thereby achieving superior performance compared with traditional purely data-driven clustering algorithms. Among these methods, the TCN is the most sensitive to measurement deviations, whereas the Transformer fails to fully exploit its potential due to the limited amount of training data.

Unlike the other methods, the Denoising Autoencoder exhibits a trend in which the sorting accuracy first increases and then decreases. This phenomenon is related to the digitization strategy adopted during data preprocessing. Specifically, the method performs equal-interval sampling based on time units. Therefore, different TOAs not exceeding a time unit can fall into one same time unit, which alleviates the interference caused by measurement errors to some extent. The sorting accuracies of the Denoising Autoencoder algorithm are similar on Dataset 1, Dataset 2, and Dataset 3, where the measurement errors are relatively small. The noticeable improvement in accuracy on Dataset 4 is attributed to the fact that the measurement error approximately exceeds the time unit, which effectively acts as a form of data augmentation. However, the accuracy decreases again on Dataset 5 because excessive measurement errors cause severe data distortion, affecting network learning.

A similar phenomenon can also be observed in U1DADM, which also uses digitized data. The sorting accuracy decreases significantly on Dataset 4, where the intra-pulse parameters exhibit severe dispersion and overlap. Nevertheless, TOA digitization provides a certain denoising effect for inter-pulse information, and thus the sorting accuracy degradation becomes more pronounced on Dataset 5. U1DADM learns both intra-pulse and inter-pulse parameter information, thus achieving the highest sorting accuracy and strongest robustness compared to other methods. Furthermore, analysis of the experimental results on Dataset 6 and Dataset 7 with combined measurement errors shows that the network can still achieve relatively high sorting accuracy as long as either the intra-pulse features or the inter-pulse features remain sufficiently distinguishable. Moreover, inter-pulse information is more robust than intra-pulse information, primarily due to the enhanced noise resistance introduced by the digitization process.

The proposed U1DADM, TCN, and transformer achieve the best performance, with both the SOGR and SUGR equal to zero. In contrast, the SOGR of Improved K-Means gradually increases due to parameter dispersion, and the SUGR of DBSCAN gradually increases because of parameter-range overlap. In addition, the proposed U1DADM also exhibits advantages over the other deep learning methods in terms of memory consumption and computational time.

### 4.5. More Experiments and Analysis

(1) The impact of false pulses and missing pulses on U1DADM

**Figure 7:**
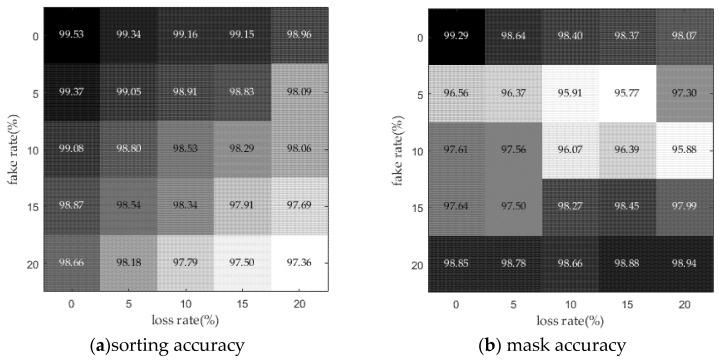
Sorting accuracy and mask accuracy at different pulse loss rates and false pulse rates.

To further demonstrate the robustness and anti-interference capability of the proposed U1DADM network in complex electromagnetic environments, datasets with different proportions of false pulses and missing pulses were constructed based on Dataset 1 to simulate real electromagnetic propagation environments. Experiments were conducted on these datasets, and the corresponding results are shown in Figure 7.

The figure shows that the sorting accuracy is highest when there are no pulse loss or false pulses. As either the pulse missing rate or the false pulse rate increases, the sorting accuracy decreases to varying degrees. This result is consistent with the physical characteristics of RSS, because missing pulses lead to incomplete radar feature information, while false pulses introduce additional interference. Both factors adversely affect the extraction and learning of the inter-pulse feature patterns. It is worth noting that the sorting accuracy decreases more rapidly with the increase of the false pulse rate than with the increase of the missing pulse rate. This is because false pulses possess characteristics similar to those of genuine pulses and are therefore more likely to be identified as real pulses, resulting in false PRI patterns that interfere with the discrimination capability of the network. Finally, it can be observed that the proposed U1DADM network still maintains a sorting accuracy above 97% even under the worst-case condition, where both the pulse missing rate and the false pulse rate reach 20%. This demonstrates that the U1DADM network possesses redundancy and fault tolerance in feature extraction, thereby exhibiting strong robustness in complex electromagnetic environments.

The mask accuracy exhibits a distribution pattern different from that of the sorting accuracy, showing noticeable fluctuation. This phenomenon arises because both pulse loss and the presence of false pulses affect the pulse overlap conditions. The accuracy exhibits a trend of first decreasing and then increasing as the false pulse rate increases. The initial decrease is caused by the increased pulse overlap risk introduced by false pulses, which interferes with the mask prediction process. However, as the number of false pulses further increases, the network gradually improves its capability to learn and distinguish such interference. By adaptively enlarging the dilation rates of ADC, the network becomes capable of better capturing the characteristics of genuine pulses, thereby resulting in a subsequent improvement in sorting accuracy. The variation trend of mask accuracy with increasing pulse missing rate is more complex. Specifically, when no false pulses are present, the mask accuracy decreases steadily. When the false pulse rate is 5% or 20%, the mask accuracy first decreases and then increases, whereas for false pulse rates of 10% and 15%, the accuracy first decreases, then increases, and finally decreases again. In the absence of false pulses, the number of pulse overlaps decreases as the number of pulses decreases, resulting in fewer learnable masks and thus lower accuracy. When there are fewer false pulses, the overlap between real and false pulses interferes with mask learning; however, as the number of genuine pulses decreases, the overlap with false pulses is correspondingly reduced, resulting in a partial recovery of mask accuracy. For false pulse rates of 10% and 15%, the final decrease in mask accuracy is caused by the increasing proportion of false pulses within the overall pulse stream, which further interferes with the prediction results. In contrast, when the false pulse rate reaches 20%, the mask accuracy no longer decreases at the final stage because the number of false pulses becomes sufficiently large for the network to effectively learn their characteristics and distinguish them from genuine pulses. But in either case, the mask accuracy rate remains above 95%, demonstrating its effectiveness in assisting the pulse sorting process.

(2) The Influence of Pulse Stream Density and Time Unit Selection on U1DADM

To further investigate the effects of the digitization time unit and pulse stream density on the pulse overlap rate and sorting performance, Dataset 1 is used as the baseline. Specifically, the PRI of each RRS is decreased by 10% and increased by 10%, respectively, to construct datasets with different pulse stream densities. Meanwhile, three digitization time units, namely 2 μs, 5 μs, and 10 μs, are adopted to digitize each dataset, thereby generating different levels of pulse overlap in the digitized PDW.

By statistically analyzing the pulse overlap rates under different experimental conditions and evaluating their impact on sorting performance, the effectiveness and robustness of the proposed U1DADM network under varying pulse stream densities and pulse overlap levels are verified. The pulse overlap rates of the digitized PDW under different time unit settings and pulse stream densities are presented in Table 8, while the corresponding sorting accuracies and mask accuracies are reported in Table 9 and Table 10.

As shown in Table 8, the pulse overlap rate of the digitized PDW increases with both the pulse stream density and the time unit. This is because a higher pulse stream density results in more pulses arriving within a given time interval, while a larger digitization time unit maps more pulses into the same discrete time unit. Consequently, both factors increase the probability of pulse overlap.

As for the sorting performance, the sorting accuracy exhibits a slight downward trend as the pulse overlap rate increases. This indicates that a higher pulse overlap rate aggravates the mixing of pulses from different RRS, thereby increasing the difficulty of pulse sorting. Nevertheless, even when the pulse overlap rate reaches 53.12%, the proposed U1DADM still achieves a sorting accuracy of over 98.98%, demonstrating its strong robustness and stability under high-density pulse stream conditions.

On the other hand, the mask accuracy consistently remains above 99.2% and exhibits a slight improvement as the pulse overlap rate increases. This can be attributed to the larger number of overlapping pulse samples available during both training and testing, which provides richer supervision for the mask prediction branch. Consequently, the network is able to learn the characteristics of overlap regions more effectively, leading to improved mask prediction performance. In contrast, when the pulse overlap rate is relatively low, the number of overlapping samples is limited, restricting the learning of overlap patterns and resulting in a slight decrease in mask accuracy.

Overall, the experimental results demonstrate that both the time unit and the pulse stream density directly affect the pulse overlap rate of the digitized PDW, which in turn influences the difficulty of pulse sorting. Although the sorting performance degrades slightly as the pulse overlap rate increases, the proposed U1DADM consistently maintains high sorting and mask prediction accuracies. These results demonstrate that the proposed data, proposed method, masking assistance strategy, and adaptive dilated convolution effectively adapt to complex scenarios with different pulse stream densities and overlap levels, exhibiting strong robustness and generalization capability.

## 5. Conclusions

This paper proposes a novel RSS method for scenarios involving identical modulation types and overlapping parameter ranges. For the digitized PDW data obtained after preprocessing, a one-dimensional neural network termed U1DADM is designed based on the concept of semantic segmentation. In addition, a mask-assisted strategy is introduced to enhance the network’s ability to perceive and recognize overlapping pulses within the same time unit.

Regarding data preprocessing, the proposed digitized PDW representation method preserves the complete information of intra-pulse and inter-pulse features while unifying the input data length. Moreover, it effectively improves the non-stationarity and non-convergence issues caused by the continuous temporal growth of TOA data, thereby enhancing the stability of network training and the capability of feature learning.

In terms of network architecture design, the ADC module in U1DADM dynamically adjusts the dilation coefficient to adaptively match the sparsity characteristics of different pulse data, thus flexibly adjusting the network receptive field. Simultaneously, a multi-scale feature fusion mechanism enhances the modeling capability for long-range correlated features, enabling the network to capture deep semantic relationships within complex pulse streams. The down-sampling modules are employed to further enlarge the receptive field and extract abstract semantic information in higher-level feature space. The up-sampling modules progressively restore feature resolution and pulse position information, while skip connections effectively mitigate the loss of local detail information during down-sampling by integrating shallow and deep features.

As for the masking assistance strategy design, considering that multiple pulses may overlap within the same time unit in digitized PDW data and are difficult for the network to accurately distinguish, pulse-overlap mask information is introduced to assist model training. This strategy enhances the network’s capability to perceive overlapping pulse regions and improves pulse separation performance in complex scenarios.

Experimental results demonstrate that the proposed method achieves effective fusion of multidimensional pulse information and deep semantic feature extraction, thereby enabling accurate radar signal sorting under identical modulation types and overlapping parameter ranges. Furthermore, the proposed method exhibits strong generalization and robustness even under complex conditions such as measurement errors, false pulses, missing pulses, and different pulse stream density.

## Figures and Tables

**Table 1:** The confusion matrix.

		Predicted	
		1	0
true	1	TP	FN
	0	FP	TN

**Table 2:** Dataset parameter settings.

Title 1	Radar1	Radar2	Radar3	Radar4	Radar5
PW (μs)	2, 2.06, 2.11, 2.86, 3.06, 4.56, 15.8, 18.54	7.2, 7.5, 7.8, 8.8, 9.2, 10.8, 11.8, 13.2	6.7, 7, 7.3, 8.3, 8.7, 10.3, 11.3, 12.7	3.5, 4, 4.7, 5.6, 7.6	9.4, 12.9, 13.7, 16.6
RF (MHz)	3, 5, 7, 9, 11, 13, 15, 17	12, 14, 16, 18, 20, 22, 24, 26	10, 13, 16, 19, 22, 25, 28, 31	25, 29, 33, 37, 41	4, 6, 8, 10

**Table 3:** The corresponding measurement errors settings for each dataset.

	Dataset 1	Dataset 2	Dataset 3	Dataset 4	Dataset 5	Dataset 6	Dataset 7
TOA (μs)	0.25	0.5	1	2	4	4	0.25
RF (MHz)	0.5	1	2	4	8	0.5	8

**Table 4:** Sorting accuracy of different networks.

Network	Sorting Accuracy
U1D	81.61
U1DD	82.82
U1DAD (softmax)	84.06
U1DAD (sigmoid)	98.83
U1DADM	99.53

**Table 5:** Sorting accuracy of different methods. The best result is bolded, and the second best is underlined.

	Dataset 1	Dataset 2	Dataset 3	Dataset 4	Dataset 5	Dataset 6	Dataset 7
U1DADM	**99.53**	**99.35**	**99.26**	**97.82**	**92.5**	96.33	99.32
TCN	93.28	81.95	80.87	80.1	70.64	76.89	92.63
transformer	72.46	70.44	65.49	60.03	51.68	61.13	63.82
BLSTM	76.97	74.96	71.1	69.6	59.24		
autoencoder	76.82	78.45	78.6	83.05	77.62		
Improved K-Means	91.31	90.36	86.81	77.79	66.28		
DBSCAN	79.75	69.74	56.7	56.42	56.42		
SDIF	90.59	88.05	82.56	75.21	68.95		

**Table 6:** The SOGR and SUGR of different methods.

	Dataset 1	Dataset 2	Dataset 3	Dataset 4	Dataset 5
U1DADM	0/0	0/0	0/0	0/0	0/0
TCN	0/0	0/0	0/0	0/0	0/0
transformer	0/0	0/0	0/0	0/0	0/0
Improved K-Means	100/0	100/0	166/0	233/0	166/0
DBSCAN	0/33	0/33	0/66	0/66	0/66
SDIF	1466/0	2000/0	2166/0	2100/0	2000/0

**Table 7:** Computational complexity of different methods.

	Parameter Count	FLOPs (M)	Training Time (ms)	Inference Time (ms)	Memory Usage (MB)
U1DADM	9,938,015	11,252.93	18.30	4.97	189.19
TCN	1,461,958	13,051.73	16.24	4.19	247.4
transformer	796,038	6361.09	106.77	22.99	378.46
BLSTM	2,110,470	3.69	62.61	26.06	111.29
autoencoder	534,896	0.53	1.68	0.20	20.84

**Table 8:** Pulse overlap rates of the digitized PDW under different time units and pulse stream densities.

	Time Unit of 2 μs (%)	Time Unit of 5 μs (%)	Time Unit of 10 μs (%)
Dataset 1	11.71	27.29	48.79
Dataset 1 (90% PRI)	12.18	30.14	53.12
Dataset 1 (110% PRI)	10.69	25.22	45.65

**Table 9:** Sorting accuracy under different time units and pulse stream densities.

	Time Unit of 2 μs (%)	Time Unit of 5 μs (%)	Time Unit of 10 μs (%)
Dataset 1	99.78	99.53	99.1
Dataset 1 (90% PRI)	99.74	99.49	98.98
Dataset 1 (110% PRI)	99.79	99.55	99.06

**Table 10:** Mask accuracy under different time units and pulse stream densities.

	Time Unit of 2 μs (%)	Time Unit of 5 μs (%)	Time Unit of 10 μs (%)
Dataset 1	98.91	99.29	99.36
Dataset 1 (90% PRI)	99.24	99.33	99.39
Dataset 1 (110% PRI)	99.22	99.29	99.33

## Data Availability

The original contributions presented in this study are included in the article. Further inquiries can be directed to the corresponding author.
